# Supplementary material for: Neuronal microstructural changes in the human brain are associated with neurocognitive aging
Source: bioRxiv. 2024 Jan 12:2024.01.11.575206. Preprint. [Version 1] doi: 10.1101/2024.01.11.575206 (PMC10802615; doi:10.1101/2024.01.11.575206)
Supplement: Supplement 1 [file media-1.pdf]

Supplementary Information: Neuronal microstructural changes in the human brain are associated with neurocognitive aging

Kavita Singh[1*], Stephanie Barsoum[1], Kurt G Schilling[2], Yang An[3], Luigi Ferrucci[4], Dan Benjamini[1*]

Affiliations:

[1]Multiscale Imaging and Integrative Biophysics Unit, National Institute on Aging, NIH, Baltimore, MD, USA

[2]Department of Radiology and Radiological Sciences, Vanderbilt University Medical Center, Nashville, TN, USA

[3]Brain Aging and Behavior Section, National Institute on Aging, NIH, Baltimore, MD, USA

[4]Translational Gerontology Branch, National Institute on Aging, NIH, Baltimore, MD, USA

*Correspondence to:
Dan Benjamini, PhD
The National Institute on Aging
251 Bayview Blvd. Baltimore, MD 21224, USA
Email: dan.benjamini@nih.gov

Correspondence may also be sent to:
Kavita Singh, PhD
The National Institute on Aging
251 Bayview Blvd. Baltimore, MD 21224, USA
Email: kavita.singh3@nih.gov

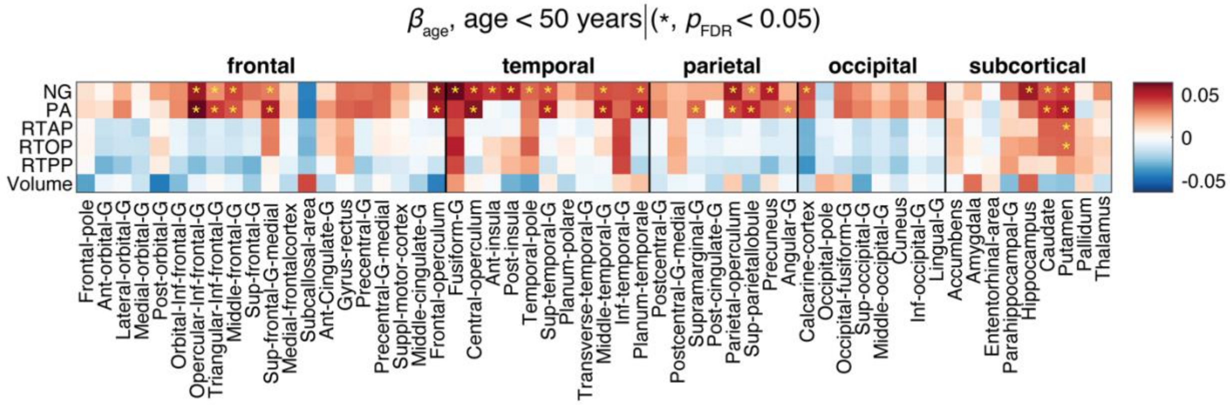

Supplementary Figure 1: Linear associations of MAP-MRI and volumetric metrics and age in a subset of subjects under the age of 50 (N=227). The β_{age} coefficients are shown as a matrix for MAP-MRI and volumetric z-normalized features across all 56 ROIs. Blocks marked with an asterisk (*) represent associations meeting the $p_{\text{FDR}}<0.05$ threshold.
